# Glucagon-like peptide-1 receptor agonists for the treatment of opioid use disorders: a systematic review

**DOI:** 10.1017/neu.2025.10038

**Published:** 2025-09-03

**Authors:** Hezekiah C.T. Au, Pak Ho Lam, Fateen Kabir, Chen Lily Huang, Christine E. Dri, Gia Han Le, Angela T.H. Kwan, Sabrina Wong, Kayla M. Teopiz, Roger S. McIntyre

**Affiliations:** 1 Institute of Medical Science, University of Toronto, Toronto, ON, Canada; 2 Brain and Cognition Discovery Foundation, Toronto, ON, Canada; 3 Institute of Epidemiology and Health Care, University College London, London, UK; 4 Mood Disorders Psychopharmacology Unit, Toronto, ON, Canada; 5 Faculty of Medicine, University of Ottawa, Ottawa, ON, Canada; 6 Department of Pharmacology & Toxicology, University of Toronto, Toronto, ON, Canada; 7 Department of Psychiatry, University of Toronto, Toronto, ON, Canada

**Keywords:** Exenatide, liraglutide, glucagon-like peptide-1, opioid use disorder, substance use disorder

## Abstract

**Introduction:**

Extant literature indicated that glucagon-like peptide-1 (GLP-1) and glucagon-like peptide-1 receptor agonists (GLP-1 RAs) may potentially reduce risk of opioid overdose in persons with opioid use disorders (OUDs). Herein, we conducted a comprehensive synthesis of the effects of GLP-1 and GLP-1 RAs on OUDs.

**Methods:**

We examined preclinical and clinical paradigms examining the effects of GLP-1 and GLP-1 RAs on OUD and OUD-associated behaviours (i.e. opioid self-administration, opioid-seeking behaviour). Relevant articles were retrieved from OVID (MedLine, Embase, AMED, PsychINFO, and JBI EBP Database), PubMed, and Web of Science from database inception to 1 May 2025. Primary studies (*n* = 10) examining the aforementioned effects associated with GLP-1 and GLP-1 RA administration were retrieved for analysis.

**Results:**

GLP-1 RAs (i.e. exenatide, liraglutide) reduced opioid-seeking behaviour (*p* < 0.05) and self-administration of opioid drugs (*p* < 0.05) in preclinical paradigms. In addition, results from human studies indicate that GLP-1 administration was associated with reducing the risk of opioid overdose in human studies (aIRR = 0.60, 95% CI [0.43, 0.83]).

**Conclusion:**

GLP-1 RAs may affect opioid self-administration as well as the risk for overdose as evidenced by both preclinical and clinical data. There is a need for adequate well-controlled studies to determine whether GLP-1 RAs may provide clinically meaningful improvement and risk reduction in persons living with OUDs.

## Summations

- Glucagon-like peptide-1 receptor agonism was associated with reduced risk of opioid overdose in humans.
- Glucagon-like peptide-1 receptor agonism was associated with reduced heroin-seeking behaviour.
- Glucagon-like peptide-1 receptor agonism was associated with reducing opioid self-administration.

## Considerations

- No clinical trials are completely completed, limiting the ability to establish casual relationships.
- Findings from animal studies may not be consistently extended to humans.
- The specific molecular and cellular pathways wherein this interaction can be observed remains to be elucidated.

## Introduction

Glucagon-like peptide-1 (GLP-1) is an incretin hormone derived from the gut, with robust efficacy as an antidiabetic and antiobesity therapy. GLP-1 increases insulin production and exocytosis while inhibiting glucagon secretion (Lutz and Osto, 2016). GLP-1 receptors are broadly distributed peripherally (i.e. pancreas, liver) and within the central nervous system, including regions such as the hypothalamus, hippocampus, and nucleus accumbens (Muscogiuri *et al*., 2017).

Opioid use disorder (OUD) is a severe and persistent disorder characterised by maladaptive self-administration of drugs that agonise opioid receptors, notably the μ opioid receptor (μOR) (Sharma *et al*., 2016). Chronic consumption of opioid-related drugs progressively disrupts the function of multiple neurotransmitters, especially dopaminergic neurons via reduced firing of inhibitory GABAergic neurons leading to dysregulation in dopamine signalling in the mesolimbic region, which subserves the pathophysiology of addictive disorders (Margolis *et al*., 2003; Baik, 2013).

A growing body of observational, preclinical and clinical evidence has implicated GLP-1 RAs as putative prevention and/or treatment strategies in persons living with neurocognitive disorders (McIntyre *et al*., 2025; Au *et al*., 2025. Recently, GLP-1 receptor agonists (GLP-1 RAs) have been implicated as a potential treatment strategy for substance use disorders (SUDs), including OUDs (Bruns *et al*., 2024; Lee *et al*., 2024; Mansur *et al*., 2024; Zheng *et al*., 2025). Notably, stimulation of GLP-1 receptors in GABAergic interneurons in the ventral tegmental area (VTA) has been shown to modulate dopamine release by decreasing activity of VTA dopaminergic neurons (Merkel *et al*., 2025). Whether GLP-1 RAs meaningfully affect craving, consumption, withdrawal, reinforcement and/or overdose in persons with OUD is not fully known. In addition, it has been reported that GLP-1 RAs may be adequately penetrative of the central nervous system, suggesting their effects might be a direct effect (West *et al*., 2025).

Herein, we examine the effects of GLP-1 RAs (i.e. dulaglutide, exenatide, liraglutide, lixisenatide, and semaglutide) on the modulation of OUD-associated behaviours (e.g. opioid seeking, self-administration) in preclinical and clinical studies. The overarching aim is to provide a compelling rationale for evaluating GLP-1 RAs as potential treatment and/or prevention strategies in persons living with OUDs.

## Methods

### Search strategy

The Preferred Reporting Items for Systematic Reviews and Meta-Analyses (PRISMA) 2020 guidelines were used to conduct this review (Page *et al*., 2021). PubMed, Web of Science, and OVID (MedLINE, Embase, AMED, PsychInfo, and JBI EBP) were searched systematically for articles from database inception to May 1st, 2025. The search string used for the search was: (“GLP-1” OR “Glucagon-Like Peptide-1” OR “Glucagon-Like Peptide 1” OR “GLP-1 Agonist” OR “Glucagon-Like Peptide-1 Agonist” OR “Glucagon-Like Peptide 1 Agonist” OR “Semaglutide” OR “Ozempic” OR “Rybelsus” OR “Wegovy” OR “Dulaglutide” OR “Trulicity” OR “Exenatide” OR “Byetta” OR “Bydureon” OR “Liraglutide” OR “Lixisenatide”)AND (“Substance*” OR “Opioid*” OR “Opiu*” OR “Codeine” OR “Morphine” OR “Oxycodone” OR “Hydrocodone” OR “Dihydrocodein*” OR “Hydromorphone” OR “Diamorphine” OR “Fentanyl” OR “Heroin”).

### Study selection and inclusion criteria

Articles from the literature search were systematically screened via the Covidence platform, wherein duplicate articles were removed (Covidence 2024). Two reviewers (H.A. and P.H.L.) screened the titles and abstracts based on the inclusion and exclusion criteria (Table 1). Primary articles were retrieved for full-text screening by both reviewers (H.A. and P.H.L.) if they reported on the effects of opioid consumption and overdose risk following GLP-1 or GLP-1 RA administration.

**Table 1.** Eligibility criteria

Inclusion Criteria	A primary study, Human Studies, Patients must be between the ages 18-65 (adults), Measurement of GLP-1 secretion, Measurement of opioid usage, Full-text article available online, English language.
Exclusion Criteria	Non-primary or secondary research (i.e., literature reviews, systematic reviews, meta-analyses, posters, abstracts, guidelines, protocols and theses), Case Studies, Reports an association without statistics, Full-Text is not available.

### Data extraction

Relevant information was extracted and organised using the piloted data extraction template by two independent reviewers (H.A. and P.H.L.). Information of interest to be extracted was established *a priori*for human studies, including (1) authors, (2) study type, (3) sample size, (4) diagnoses, (5) mean age, and (6) outcome of interest. Similarly, information of interest was established *a priori* for animal studies, including (1) authors, (2) study type, (3) sample size, (4) diagnoses, and (5) outcome of interest. Outcomes of interest pertained to changes in opioid overdose risk, opioid self-administration, and consumption following administration of GLP-1 and GLP-1 RAs.

### Quality assessment

Quality assessment of observational cohort studies was conducted using the Quality Assessment for Observational Cohort and Cross-Sectional Studies from the National Institute of Health (Ma *et al*., 2020; National Institute of Health, 2013). Similarly, quality assessment for animal studies was conducted using the SYRCLE’s risk of bias analysis tool for animal studies (Hooijmans *et al*., 2014). The risk of bias of all studies were assessed by two independent reviewers (F.K., P.K.L.), wherein all conflicts were resolved following discussion. Further information on the methodological quality assessments has been listed in the supplementary materials (Tables S1 and S2).

## Results

### Search results

**Figure 1.**
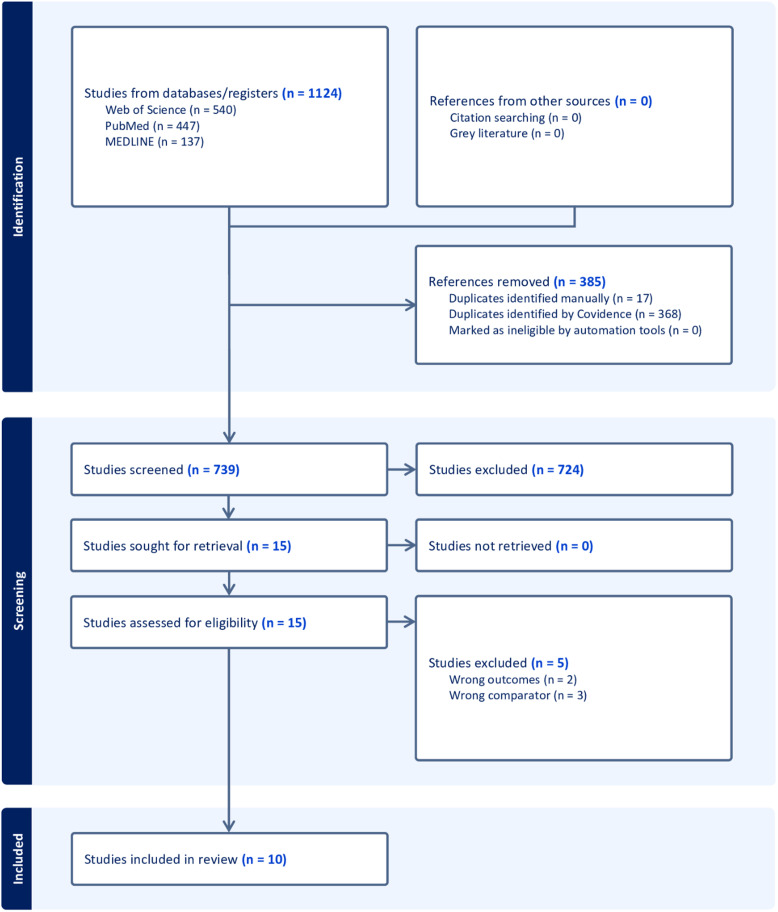
PRISMA flow diagram of literature search (Covidence 2024).

A systematic search retrieved 1,124 studies. 368 duplicates were removed through Covidence, and 17 duplicates were removed manually. 739 studies underwent abstract and title screening, wherein 15 relevant full-text articles were retrieved and screened based on the inclusion and exclusion criteria (Table 1). Five studies were excluded as a result of wrong outcomes (*n* = 2) or wrong comparators (*n* = 3), characterised by inclusion of results irrelevant to OUD, and a lack of comparison to control groups respectively. A total of 10 studies were retrieved for further analysis (Fig. 1).

### Methodological quality

The study by Qeadan *et al*. (2025) clearly stated its research question, defined exposure and outcome variables using reliable sources, and adjusted for key confounders such as comorbidities and sociodemographic variables. While it did not report blind outcome assessors to exposure status, they demonstrated low risk of bias overall. Limitations included lack of repeated exposure measurement and absence of power or sample size justifications. Nonetheless, these issues were unlikely to substantially affect the validity of the main findings.

Most preclinical studies reported similar baseline characteristics across groups, addressed incomplete outcome data appropriately, and avoided selective outcome reporting. Frequent methodological shortcomings included lack of reported randomisation procedures, allocation concealment, blinding of investigators, and outcome assessors. These limitations were consistently observed across most studies, limiting internal validity. All publications had a low risk of bias for other domains.

### Effects of GLP-1 RAs on opioid use disorders in humans

There is currently insufficient information with regard to the association between GLP-1 administration and risk of OUDs (Table 2). Notwithstanding, a retrospective cohort study conducted by Qeadan *et al*. (2025) reported that GLP-1 administration was associated with lowered levels of opioid overdose in individuals with OUD (7.1% vs 15.7%). When comparing between disease-based cohorts, individuals with diabetes and OUD exhibited a lower rate of opioid overdose following GLP-1/GIP administration (aIRR = 0.62, 95% CI [0.46, 0.82]) (Qeadan *et al*., 2025). These findings were replicated in individuals with obesity and OUD (aIRR = 0.67, 95% CI [0.49, 0.92]), and individuals with diabetes, obesity, and OUD (aIRR = 0.65, 95% CI [0.48, 0.88]) (Qeadan *et al*., 2025). Evidently, further research is required to elucidate the mechanism and effects of various GLP-1 RAs on OUDs in humans.

**Table 2.** Study characteristics investigating effect of GLP-1 agonists on opioid use disorders in human models

Author(s)	Study type	Sample size	Diagnoses	Mean age	Outcome(s) of interest
**Glucagon-like peptide-1**					
Qeadan *et al*. (2025)	Retrospective Cohort Study	503,747 participants with history of OUD	T2DM Obesity OUD	**OUD:** 50.5 ± 18.1 years	GLP-1 administration was associated with lowered levels of opioid overdose in individuals with OUD (7.1% vs 15.7%). Individuals with OUD that are administered with GLP-1/GIP had a 40% lower rate of opioid overdose in comparison to those without (aIRR = 0.60, 95% CI [0.43, 0.83]). These trends were replicated in individuals with diabetes and OUD (aIRR = 0.62, 95% CI [0.46, 0.82]), individuals with obesity and OUD (aIRR = 0.67, 95% CI [0.49, 0.92]), and individuals with diabetes, obesity, and OUD (aIRR = 0.65, 95% CI [0.48, 0.88]).

Abbreviations: N/A = Not Available, T2 DM = Type 2 Diabetes Mellitus, OUD = Opioid Use Disorder.

### Effects of GLP-1 RAs on opioid use disorders in animal models

We identified 9 studies that reported on the effect of disparate GLP-1 RAs on opioid self-administration and seeking behaviour in animal models.

#### Effects of exenatide on opioid use disorders in animal models

Bornebusch *et al*. (2019) examines the effect of exenatide on the self-administration of remifentanil – a synthetic opioid drug. They reported that administration of 10 µg/kg exendin-4 (Ex-4) did not significantly affect the rate of acquisition or number of remifentanil reinforcers in B6 mice (*p* > 0.05) (Bornebusch *et al*., 2019). In contrast, however, a study by Douton *et al*. (2021a) reported that administration of 2.4 µg/kg Ex-4 increased the latency to first contact (*p* < 0.05) and contacts (*p* < 0.05) with the active spout for heroin in comparison to vehicle administration (*n* = 24). Notwithstanding, acute treatment of Ex-4 six hours prior to drug-induced reinstatement tests did not affect heroin-seeking behaviour (*p* > 0.05) (Douton *et al*., 2021a). This suggests that there may be possible dosage-related effects that may affect exenatide effects on heroin-seeking behaviour and self-administration.

A separate study by Zhang *et al*. (2020a) reported that systemic administration of Ex-4 decreased motivation to self-administer oxycodone (*n* = 20; *p* < 0.05). Additionally, Ex-4 pre-treatment directly to the nucleus accumbens shell attenuated oxycodone self-administration (*p* < 0.05) and cue-induced reinstatement of drug-seeking behaviour (*p* < 0.05) (Zhang *et al*., 2020a). These findings were replicated in a separate study by Zhang *et al*. (2021), wherein administration of Ex-4 decreased frequency of fentanyl self-administration and active lever responses (*n* = 27; *p* < 0.05). Additionally, Ex-4 significantly attenuated reinstatement of fentanyl-seeking behaviour (*n* = 13; *p* < 0.05) (Zhang *et al*., 2021). Taken together, these findings suggest that administration of exenatide reduces opioid self-administration and may improve opioid-seeking behaviours.

#### Effects of liraglutide on opioid use disorders in animal models

We identified 5 studies that examined the effects of liraglutide on opioid self-administration and seeking behaviour in animal models (Table 3). Findings by Urbanik *et al*. (2022) reported that liraglutide administration was associated with significantly reduced cue-induced fentanyl seeking behaviour in comparison to saline administration (*p* < 0.05). These trends were replicated in both low drug takers (*n* = 10; *p* < 0.05) and high drug takers (*n* = 14; *p* < 0.05). A separate study by Urbanik *et al*. (2025) reported similar trends in female Sprague-Dawley rats, wherein pre-treatment of liraglutide was associated with reduced drug-induced fentanyl-seeking behaviour (*p* = 0.0001) during reinstatement tests. These findings were replicated in low drug takers at hour 1 of the experiment (*p* = 0.0499), but not at hour 4 (*p* = 0.4532), and in high drug takers at hour 4 of the experiment (*p* < 0.05), but not at hour 1 (*p* = 0.7109) (Urbanik *et al*., 2025). These findings were further supported by a separate study by Evans *et al*. (2022) (*n* = 52), wherein liraglutide administration was associated with attenuating self-administration of heroin in low heroin takers (*F*_3,30_ = 20.70, *p* < 0.0001) and high heroin takers (*F*_3,33_ = 11.99, *p* < 0.0001) over time. Additionally, liraglutide also reduced infusion attempts in comparison to vehicle administration (*p* < 0.05) (Evans *et al*., 2022).

**Table 3.** Study characteristics investigating effect of GLP-1 agonists on opioid use disorders in animal models

Author(s)	Study type	Sample size	Reinstatement stimuli	Outcome(s) of interest
**Exenatide**				
Bornebusch *et al*. (2019)	Animal study	Male B6 mice	Ethanol self-administration Remifentanil self-administration	10 µg/kg Ex-4 was administered to mice. Administration of Ex-4 did not significantly affect the rate of acquisition or number of remifentanil reinforcers (*p* > 0.05).
Douton *et al*. (2021a)	Animal study	55 male Sprague- Dawley rats	Cue-Induced Heroin Seeking Reinstatement Drug-Induced Heroin Seeking Reinstatement	Rats were administered daily with 2.4 µg/kg Ex-4 (*n* = 25) or vehicle (*n* = 24). Administration of Ex-4 significantly increased the latency to first contact (*p* < 0.05) and contacts (*p* < 0.05) with the active spout for heroin in comparison to vehicular treatment. Treatment of Ex-4 six hours prior to drug-induced reinstatement tests did not affect drug-induced heroin seeking behaviour (*p* > 0.05).
Zhang *et al*. (2020a)	Animal study	Male Sprague- Dawley rats	Oxycodone self- administration Cue-Induced Oxycodone-Seeking Reinstatement	Rats were systemically administered with Ex-4 (0.3 and 3.0 µg/kg) or pretreated (0.005 or 0.05 µg) 10 minutes prior to each trial. Systemic administration of Ex-4 (*n* = 20) at 0.3 and 3.0 µg/kg decreased motivation to self-administer oxycodone (*p* < 0.05), characterised by decreased total oxycodone infusions prior to reaching a specific threshold on a progressive-ratio schedule of reinforcement. Similarly, pretreatment of Ex-4 (0.005 or 0.05 µg) in the nucleus accumbens shell attenuates oxycodone self-administration (*p* < 0.05) and cue-induced reinstatement of drug-seeking behavior (*p* < 0.05).
Zhang *et al*. (2021)	Animal study	Male Sprague- Dawley rats	Fentanyl self-administration Cue-Primed Reinstatement	Administration of 0.72 nmol/kg exendin-4 decreased levels of fentanyl self-administration and active lever responses (*n* = 27; *p* < 0.05), but not at 0.072 nmol/kg exendin-4 (*n* = 27; *p* > 0.05). Administration of 0.072 nmol/kg (*n* = 13) and 0.72 nmol/kg (*n* = 13) exendin-4 significantly attenuated reinstatement of fentanyl-seeking behavior (*p* < 0.05). The effect was more pronounced in rats administered with 0.72 nmol/kg exendin-4, although this relationship was insignificant.
**Liraglutide**				
Douton *et al*. (2022)	Animal study	63 male Sprague- Dawley rats	Cue-Induced Heroin Seeking Reinstatement Drug-Induced Heroin Seeking Reinstatement	Rats were administered with 0.06 mg/kg liraglutide daily for three days, ramping up to 0.3 mg/kg liraglutide daily for three days, and up to 1.0 mg/kg liraglutide for three more days. Rats were pre-treated with 0.3 mg/kg liraglutide 6 hours prior to extinction and reinstatement of heroin-seeking behaviour. Rats that were treated with liraglutide exhibited significant decreases in cue-induced seeking behaviour during extinction (*p* < 0.05) and drug-induced reinstatement of heroin seeking (*p* < 0.05). A significant group × pretreatment × time interaction was observed (F_2,36_ = 7.07; *p* < 0.005), wherein vehicle-treated rats with a history of heroin self-administration exhibited greater heroin-seeking behaviour than heroin-liraglutide-treated controls in hour 1, wherein extinguished seeking behaviour was observed by hour 3. Additionally, rats treated with liraglutide made significantly fewer contacts with active spouts for heroin self-administration in comparison to vehicle treated rats (*p* < 0.001). These trends were replicated following 14 days of abstinence, wherein liraglutide pretreatment was associated with reduced cue-induced heroin seeking behavior (*p* < 0.05). Additionally, liraglutide reduced spout contacts in comparison to vehicle administration (*p* < 0.05), whilst simultaneously reducing stress-induced reinstatement of heroin seeking behaviour (*p* < 0.05).
Douton *et al*. (2021b)	Animal study	95 male Sprague- Dawley rats	Cue-Induced Heroin Seeking Reinstatement Drug-Induced Heroin Seeking Reinstatement	Rats were administered daily with 0.1 mg/kg liraglutide or vehicle. Liraglutide administration was associated with increased time to contact with the heroin-associated spout (*p* < 0.05). Additionally, liraglutide consistently decreased self-administration of heroin in each trial, but the effect of treatment was significant only after hour 5 (between hours 1-6) of the trial (F_1,14_ = 10.73; *p* < 0.01). Administration of liraglutide was associated with significantly lowered levels of active spout contacts in comparison to vehicle administration during drug-induced reinstatement of heroin-seeking behaviour (t(8) = 2.57; *p* < 0.05).
Evans *et al*. (2022)	Animal study	52 male Sprague- Dawley rats	Cue-Induced Heroin Seeking Reinstatement Drug-Induced Heroin Seeking Reinstatement	Rats were administered with liraglutide 0.06 mg/kg daily, ramping up to 0.1 mg/kg, 0.3 mg/kg, and 0.6 mg/kg every three days. A significant effect of liraglutide was observed over time in low heroin takers (F_3,30_ = 20.70, *p* < 0.0001) and high heroin takers (F_3,33_ = 11.99, *p* < 0.0001) over time. In cue-induced reinstatement tests, administration of liraglutide significantly reduced infusion attempts in comparison to vehicle administration (*p* < 0.05).
Urbanik *et al*. (2022)	Animal study	24 male Sprague- Dawley rats	Cue-Induced Fentanyl Seeking Reinstatement Drug-Induced Fentanyl Seeking Reinstatement	Rats readily self-administered fentanyl for 14 days, wherein some rats were low drug takers (*n* = 10) and some were high drug takers (*n* = 14). Liraglutide administration was associated with significantly fewer cue-induced fentanyl seeking behaviour in comparison to saline administration (*p* < 0.05) in both low drug takers and high drug takers.
Urbanik *et al*. (2025)	Animal study	27 female Sprague-Dawley rats	Cue-Induced Fentanyl Seeking Reinstatement Drug-Induced Fentanyl Seeking Reinstatement	Rats readily self-administered fentanyl for 9 days, wherein some rats were low drug takers (*n* = 15)and some were high drug takers (*n* = 12). Pre-treatment of 0.3 mg / kg liraglutide 6 hours prior to the experiment had no effect on fentanyl-seeking behavior at hour 1 of the experiment (*p* = 0.2166), but a reduction in drug-induced fentanyl seeking behaviour was observed at hour 4 (*p* = 0.0001). Liraglutide administration significantly reduced fentanyl seeking behaviour at hour 1 in low drug takers (*p* = 0.0499), but not significantly at hour 4 (*p* = 0.4532). Contrastingly, liraglutide did not reduce fentanyl seeking behaviour at hour 1 in high drug takers (*p* = 0.7109), but significantly reduced drug-induced fentanyl seeking behaviour at hour 4 (*p* < 0.05).

Abbreviations: N/A = Not Available, Ex-4 = Exendin-4.

In a study by Douton *et al*. (2021b) (*n* = 95), liraglutide administration was associated with increased time to contact with the heroin-associated spout (*p* < 0.05) and consistently decreasing self-administration of heroin (*F*_1,14_ = 10.73; *p* < 0.01). Additionally, liraglutide administration was associated with lowered levels of active spout contacts in comparison to vehicle administration during drug-induced reinstatement of heroin-seeking behaviour (t(8) = 2.57; *p* < 0.05) (Douton *et al*., 2021b). These trends were supported in a separate study by Douton *et al*. (2022) (*n* = 63), wherein liraglutide reduced cue-induced seeking behaviour during extinction (*p* < 0.05) and drug-induced reinstatement of heroin seeking (*p* < 0.05). Similarly, rats treated with liraglutide made fewer contacts with active spouts for heroin self-administration in comparison to vehicle treated rats (*p* < 0.05), simultaneously reducing stress-induced reinstatement of heroin seeking behaviour (*p* < 0.05) (Douton *et al*., 2022). These results suggest that liraglutide may reduce opioid-seeking behaviour and opioid consumption in both low and high drug takers.

## Discussion

To our knowledge, this review is the first study to systematically examine the effects of GLP-1 RAs on individuals and animal models of OUDs. Extant literature reports that administration of disparate GLP-1 RAs are associated with reducing risk of opioid overdose in humans, whilst attenuating opioid self-administration, opioid-seeking behaviour, and drug-induced reinstatement of opioid-seeking behaviour in animal models.

Overall, our results indicate that GLP-1 RA administration is associated with reducing self-administration of various opioids and attenuate cue-induced reinstatement of opioid-seeking behaviour. Specifically, exenatide administration also increased the latency to first contact and subsequent contacts with the heroin spout. Administration of Ex-4 directly to the nucleus accumbens also attenuated opioid-seeking behaviour in animal models, which strongly suggests that GLP-1 RA effects on opioid seeking and consumption may be modulated directly via mesolimbic reward pathways. Similarly, liraglutide administration was associated with reduced opioid-seeking behaviour and infusion attempts, further supporting the notion that GLP-1 receptor agonism may modulate improvements in OUDs via reward pathways.

Indeed, extant imaging studies has highlighted that GLP-1 receptor agonism may alter functional connectivity in the nucleus tractus solitarius, which may also affect downstream connections to the ventral tegmental area (VTA) and subsequent projections to the nucleus accumbens (Au *et al*., 2024; Blum *et al*., 2025). Additionally, GLP-1 receptors on GABAergic spiny neurons in the nucleus accumbens shell may also subserve inhibition of the reward pathway following GLP-1 RA administration (Zhang *et al*., 2020b). Given the importance of the aforementioned pathways and specifically the nucleus accumbens in modulating dopaminergic neuronal signalling, it is likely that GLP-1 RAs inhibit this pathway to attenuate various symptoms of OUD observed in this study (Nicola *et al*., 2005).

Extant studies have shown that endogenous GLP-1 signalling from GLP-1 containing neurons in the nucleus tractus solitarius may modulate substance-mediated disorders (e.g. AUD) (Jerlhag, 2018). Additionally, GLP-1 mono-agonists and GLP-1/GIP dual agonists may modulate reward pathways via different mechanisms. Notably, administration of GLP-1/GIP dual agonists has been reported to exhibit greater hypoglycaemic effects and weight loss (Inagaki *et al*., 2022). Given the complex interplay among insulinotropic effects, weight loss, and dopaminergic signalling, it is important to highlight that the GIP component of GLP-1/GIP dual agonists may also further contribute to reductions in drug-seeking behaviours (Daws *et al*., 2011; Koritzky *et al*., 2014; Grespan *et al*., 2021).

Although our review observed an association between GLP-1 receptor agonism and reduced risk of opioid overdose, no causal link between GLP-1 receptor agonism and opioid use can be established. Specifically, clinical data examining the ability of endogenous GLP-1 and GLP-1 RAs to attenuate opioid-seeking behaviour remains limited, which hinders our ability to establish causal relationships. Notably, the included studies did not directly assess the effects of specific GLP-1 RAs on OUD treatment outcomes, and there are currently no published clinical trials examining the aforementioned interaction. To our knowledge, there are currently three ongoing clinical trials that are examining the effects of GLP-1 mono-agonists and dual agonists (NCT06651177, NCT06639464, NCT06548490) expected to end around 2027, which may provide necessary information with regards to whether GLP-1 RAs can be prescribed for individuals with OUD.

Inferences and interpretations of our systematic review may be affected by methodological limitations. Our review had a relatively limited number of studies that examined the effect of GLP-1 RAs on OUD. Opioids such as oxycodone, fentanyl, heroin, and remifentanil were examined, wherein these agents have varying mechanisms of actions that may introduce heterogeneity and limit the generalisability of our findings. In addition, sex-dependent effects may also confound our findings. Furthermore, administration of GLP-1 RAs varied in dosage and frequency (i.e. acute vs. chronic administration), wherein dosage-dependent effects may impact the findings of our study. Finally, consistent positive findings from small animal studies may reflect on the presence of potential publication bias. Notwithstanding, future research should be directed to examining the effects of disparate GLP-1 RAs on OUD in larger clinical trials.

## Conclusion

GLP-1 RAs may reduce both self-administration and overdose risk as evidenced by both preclinical and clinical data, reduce the severity of OUDs and alleviate risk of opioid overdose. There is a need for adequately large and well-controlled studies to determine whether GLP-1 RAs may provide for clinically meaningful improvement and risk reduction in persons living with OUD.

## Supporting information

Au et al. supplementary material Au et al. supplementary material

## Supplementary material

The supplementary material for this article can be found at https://doi.org/10.1017/neu.2025.10038.
