# Supplementary material for: Glucagon-like peptide-1 receptor agonists for the treatment of opioid use disorders: a systematic review
Source: Acta Neuropsychiatr. 2025 Sep 3;37:e85. doi: 10.1017/neu.2025.10038 (PMC13130282; doi:10.1017/neu.2025.10038)

**SUPPLEMENTARY MATERIALS**

**Table S1.** Risk of bias/quality assessment of the included studies using the NIH Quality Assessment Tool for Observational Cohort and Cross-Sectional Studies (Ma et al., 2020; NIH, 2024).

| **Study** | **Item** | | | | | | | | | | | | | | **Quality Rating** |
| --- | --- | --- | --- | --- | --- | --- | --- | --- | --- | --- | --- | --- | --- | --- | --- |
|  | **1** | **2** | **3** | **4** | **5** | **6** | **7** | **8** | **9** | **10** | **11** | **12** | **13** | **14** |  |
| Qeadan et al. (2025) | **✓** | **✓** | **NR** | **✓** | **X** | **✓** | **✓** | **✓** | **✓** | **X** | **✓** | **NA** | **✓** | **✓** | Good |

Symbols: ✓ - yes; X - no

Abbreviations: NR = not reported; NA = not applicable

**Table S2.** Risk of bias/quality assessment of the included studies using the SYRCLE’s risk of bias tool for animal studies (Hooijmans et al., 2014).

| **Study** | **Item** | | | | | | | | | | **Quality Rating** |
| --- | --- | --- | --- | --- | --- | --- | --- | --- | --- | --- | --- |
|  | **1** | **2** | **3** | **4** | **5** | **6** | **7** | **8** | **9** | **10** |  |
| Bornebusch et al. (2019) | **✓** | **✓** | **✓** | **NR** | **NR** | **X** | **✓** | **✓** | **✓** | **✓** | Good |
| Douton et al. (2021) | **✓** | **✓** | **✓** | **NR** | **NR** | **X** | **NR** | **✓** | **✓** | **✓** | Good |
| Zhang et al. (2020) | **✓** | **✓** | **✓** | **NR** | **NR** | **X** | **X** | **✓** | **✓** | **✓** | Good |
| Zhang et al. (2021) | **✓** | **✓** | **✓** | **✓** | **✓** | **✓** | **✓** | **✓** | **✓** | **✓** | Good |
| Douton et al. (2022) | **✓** | **✓** | **✓** | **✓** | **NR** | **X** | **X** | **✓** | **✓** | **✓** | Good |
| Douton et al. (2021b) | **✓** | **✓** | **✓** | **✓** | **NR** | **X** | **X** | **✓** | **✓** | **✓** | Good |
| Evans et al. (2022) | **✓** | **✓** | **✓** | **✓** | **NR** | **X** | **X** | **✓** | **✓** | **✓** | Good |
| Urbanik et al. (2022) | **✓** | **✓** | **✓** | **✓** | **NR** | **X** | **X** | **✓** | **✓** | **✓** | Good |
| Urbanik et al. (2025) | **✓** | **✓** | **✓** | **✓** | **NR** | **X** | **X** | **✓** | **✓** | **✓** | Good |

Symbols: ✓ - yes; X - no

Abbreviations: NR = not reported; NA = not applicable

**Table S3.** Effect of GLP-1 agonists on opioid use disorders.

| **GLP-1 Agonists** | **Effects on OUDs in Humans** | **Effects on OUDs in Animals** |
| --- | --- | --- |
| Exenatide | Not specified* | **Reduced:**   - Opioid self-administration - Cue-induced reinstatement of opioid seeking behavior - Drug-induced reinstatement of opioid seeking behavior |
| Liraglutide | Not specified* | **Reduced:**   - Opioid self-administration - Cue-induced reinstatement of opioid seeking behavior - Drug-induced reinstatement of opioid seeking behavior |
| Dulaglutide | Not specified* | N/A |
| Lixisenatide | Not specified* | N/A |
| Semaglutide | Not specified* | N/A |
| Tirzepatide | **Reduced:**   - Rate of opioid overdose in individuals with diabesity, obesity, and OUD. | N/A |

Abbreviations: N/A = Not Available

*Not individually assessed for association with risk of opioid overdose.

**HIGHLIGHTS**

- GLP-1 RA administration was associated with reduced risk of opioid overdose in humans.
- GLP-1 RA administration was associated with reduced heroin-seeking behavior.
- GLP-1 RA administration was associated with reducing opioid self-administration.

**GRAPHICAL ABSTRACT**


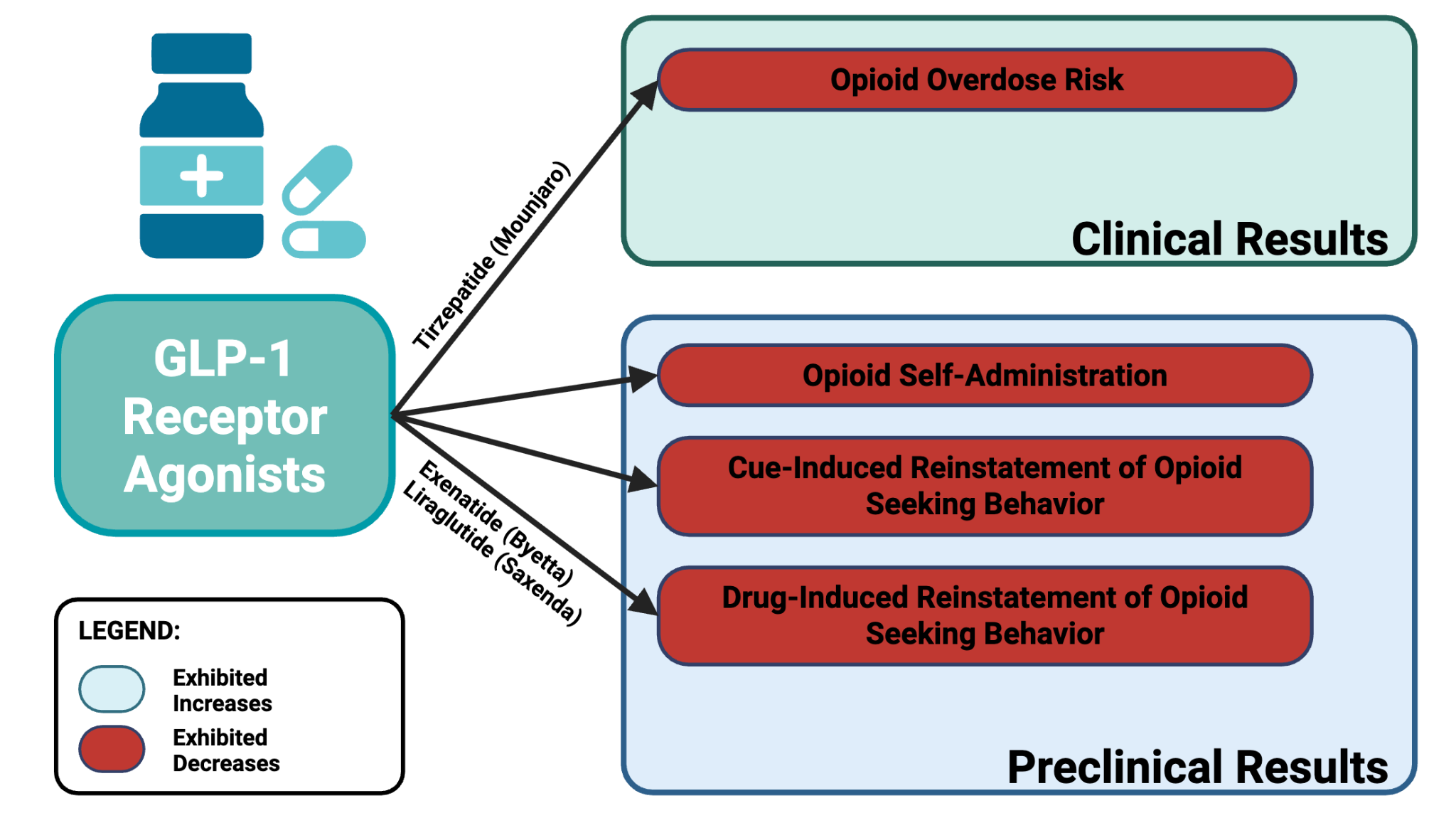

Supplement: Au et al. supplementary material [file S0924270825100380sup001.docx]
